# Deregulation of Mitochondrial ATPsyn-β in Acute Myeloid Leukemia Cells and with Increased Drug Resistance

**DOI:** 10.1371/journal.pone.0083610

**Published:** 2013-12-31

**Authors:** Xiang Xiao, Jingke Yang, Ruijuan Li, Sufang Liu, Yunxiao Xu, Wenli Zheng, Yan Yi, Yunya Luo, Fanjie Gong, Honglin Peng, Minfei Pei, Mingyang Deng, Guangsen Zhang

**Affiliations:** Division of Hematology, Institution of Molecular Hematology, The Second Xiangya Hospital, Central South University, Changsha, Hunan, P.R. China; Queen's University Belfast, United Kingdom

## Abstract

The mechanisms underlying the development of multidrug resistance in acute myeloid leukemia are not fully understood. Here we analyzed the expressions of mitochondrial ATPsyn-β in adriamycin-resistant cell line HL-60/ADM and its parental cell line HL-60. Meanwhile we compared the differences of mitochondrial ATPsyn-β expression and ATP synthase activity in 110 acute myeloid leukemia (AML, non-M3) patients between relapsed/refractory and those in remission. Our results showed that down-regulation of ATPsyn-β expression by siRNA in HL-60 cells increased cell viability and apoptotic resistance to adriamycin, while up-regulation of mitochondrial ATPsyn-β in HL-60/ADM cells enhanced cell sensitivity to adriamycin and promoted apoptosis. Mitochondrial ATPsyn-β expression and ATP synthase activity in relapsed/refractory acute myeloid leukemia patients were downregulated. This downregulated ATPsyn-β expression exhibited a positive correlation with the response to adriamycin of primary cells. A lower expression of ATPsyn-β in newly diagnosed or relapsed/refractory patients was associated with a shorter first remission duration or overall survival. Our findings show mitochondrial ATPsyn-β plays an important role in the mechanism of multidrug resistance in AML thus may present both a new marker for prognosis assessment and a new target for reversing drug resistance.

## Introduction

Acute myeloid leukemia (AML) is a clonal hematopoietic malignant disorder resulting from genetic alterations in normal hematopoietic stem cells. Although chemotherapies typically result in dramatic remissions for AML, multidrug resistance (MDR) to chemotherapy still represents a major obstacle to successful treatment, especially in relapsed or refractory patients [1]–[3]. The mechanisms of MDR include ATP-binding cassette (ABC) transporter proteins, bcl-2 family, survivin family, anti-oxidants, DNA repair activity etc. For example, multidrug resistance protein 1 (MDR1/P-glycoprotein/ABCB1) and multidrug resistance-related protein 1 (MRP1/ABCC1), both belonging to the ABC super family of membrane-bound transporters, are two genes that are found to be highly related to multidrug resistance of leukemia cells. However, the exact mechanisms identified to date in leukemia multidrug resistance have not been elucidated. Therefore, there is a need to discover new treatment strategies for relapsed/refractory AML patients.

More than half a century ago, Warburg [4] proposed that cancer cells undergo mitochondrial respiratory alterations, but this hypothesis remained largely unexplored until the presentation of “Warburg effect” in cancer biology [5] and the recent renaissance of mitochondria-mediated energy metabolism [6]–[11]. Metabolic changes are a common feature of cancerous tissues. Down-regulation of oxidative phosphorylation and concurrent activation of aerobic glycolysis is a hallmark feature of proliferating cells and of many different human cancers. In oxidative phosphorylation, the mitochondrial H^+^-ATP synthase, which is the enzyme complex of the inner mitochondrial membrane that utilizes as driving force plays an important role [12]. A decreased expression of β subunit of ATP synthase (β-F1-ATPase, ATPsyn-β), has been documented in malignant tumors when compared with its level in normal tissues. It has been consistently shown that the level of mitochondrial ATPsyn-β is significantly diminished in several solid tumors of colon, liver, kidney, esophagus, stomach, lung and breast [13]–[20]. This feature of cancer defines a ‘bioenergetic signature’ of clinical value as an indicator of disease progression as well as a predictive marker of the cellular resistance to chemotherapies [21]–[24].

Our previous finding has shown that down-regulation of mitochondrial ATPsyn-β can lead to the resistance to adriamycin in chronic myeloid leukemia (CML) [25]. In an attempt to further determine whether mitochondrial ATPsyn-β is involved in the drug resistance of AML, especially in refractory/relapsed patients, and whether ATPsyn-β is a potential target for the reversal of AML multidrug resistance, we investigated ATPsyn-β expression and mitochondrial ATPase activity in bone marrow mononuclear cells (BMMCs) and CD34^+^ cells from non-M3 AML patients. Our results suggest that deregulation of ATPsyn-β indeed plays an important role in drug resistance in AML cells. Modulation of mitochondrial ATPsyn-β may be a promising target for reversing drug resistance.

## Materials and Methods

### Ethics Statement

Investigation has been conducted in accordance with the ethical standards and according to the Declaration of Helsinki and has been approved by the Ethics Review Board of the Second Xiang-Ya Hospital, central south university. Written informed consent was obtained from all the adult patients and healthy donors analyzed. For the minors (younger than 18 years old) enrolled in the study, written informed consent was obtained from their parents.

### Cell lines and cell culture

Human acute myeloid leukemia cell line HL-60 was kindly provided by Prof. Ya Cao, Institute of Oncology, Xiang-Ya Medical School, Central South University and was routinely maintained in RPMI 1640 medium supplemented with 10% fetal bovine serum(FBS, Gibco, USA) at 37°C in a humidified atmosphere with 5%CO_2_. The drug resistant phenotype leukemia cell line HL-60/ADM was purchased from Institute of Hematology, Chinese Academy of Medical Sciences & Peking Union Medical College, which was obtained by HL-60 parental cell exposed to stepwise increasing concentrations of adriamycin that tolerates adriamycin concentrations 200 times than that of its parental HL-60 cell. Drug resistant cells were maintained in the medium containing 1.0 mg/L adriamycin(ADM, Sangon Biotech, China) and cultured in drug-free medium before they were used in the experiments.

### Patients

A total of 110 AML patients (non-M3) (female n = 56; male n = 54) and 31 healthy donors(female n = 18; male n = 13) from the Second Xiang-Ya Hospital, Central South University between April, 2009 and June, 2012 were enrolled in this study. The diagnosis of AML was made in accordance with the World Health Organization (WHO) 2008 criteria [26], [27]. Patients' characteristics are summarized in Table 1. Among them, 50 were presenting samples of AML patients who went into complete remission, 60 were from relapsed/refractory patients. Complete remission (CR) was defined by clinical and morphological criteria: the presence of 5% or less blasts in bone marrow (BM), with granulocytes>1.0×10^9^/L and platelets >100×10^9^/L. Relapse after CR was defined as the reappearance of leukemic blasts in peripheral blood or >5% blasts in BM. Patients with no CR after a double induction chemotherapy, and patients who relapsed within 6 months after CR were considered as refractory AML [28], [29]. Bone marrow specimens from patients and healthy donors were collected. BMMCs were isolated using Ficoll-Hypaque density gradient centrifugation method. The CD34 positive cells of bone marrow were isolated and purified by CD34 positive selection kit(StemCell Technologies, Canada) in 20 of AML patients (refractory n = 9, relapsed n = 3, CR n = 8) and re-suspended in RPMI 1640 supplemented with 20% fetal calf serum in an incubator.

**Table 1:** Clinical characteristic of AML patients.

Variables		No. of patients (n = 110)
	refractory/relapsed	remission duration
	(n = 50)	(n = 60)
Gender		
female	28	28
male	32	22
median age(range)	49.2years (21–75)	39.4years (15–65)
AML		
de novo	51	48
secondary	9	2
FAB classification		
M0	0	0
M1	6	4
M2	35	25
M4	8	13
M5	11	6
M6	0	2

### Cell proliferation assay

To assess cell viability after treatment with adriamycin, a MTT ((1-(4, 5-dimethylthiazol-2, 5-diphenyl) tetrazolium bromide)) assay was used. Briefly, 5×10^4^ BMMCs from AML patients (relapsed/refractory n = 42, CR n = 38) or 3×10^4^ HL-60/ADM cells and HL-60 cells were plated in triplicate wells with 200 µL RPMI 1640 medium supplemented with 10% FBS in 96-well microplates and treated with different concentrations of adriamycin. Cell viability was determined after 48 hours of continuous drug treatment. IC_50_ values were determined using a nonlinear regression program calcusyn (Biosoft, Cambridge, UK).

### Real-time PCR assay for mitochondrial ATPsyn-β gene

Total RNA was extracted from 5×10^6^ cells (including BMMCs from 110 AML patients, 31 controls; CD34^+^ cells from 20 AML patients and two cell lines) using Trizol reagent (Invitrogen, USA). Then 2 µg RNA was reverse-transcribed into cDNA using Revert Aid First Strand cDNA synthesis kit (Fermentas, USA). Transcribed cDNA was amplified using a SYBR Green I PCR reagent (Takara, Japan). Primers for mitochondrial ATPsyn-β, β-actin and MRP1 were synthesized as previously described [25], [30].

### Western blot analysis for mitochondrial ATPsyn-β protein

For protein extraction, cells (including BMMCs from 80 AML patients, 20 controls; CD34^+^ cells from 5 AML patients and cell lines) were homogenized on ice in lysis buffer (50 mM Tris-HCL, PH7.5, 150 mM NaCl, 1% NP-40, 0.25% Na-desoxycholate, 5 mM EDTA, 1 mM NaF, 25 mM Na_3_VO_4_, 0.1 mM PMSF and 2 mg/ml Aprotinin) and cellular debris was pelleted at 13 000 g for 10 min at 4°C. Equal amount of protein (80 µg/well) were separated by 8% or 10%SDS-PAGE and transferred onto PVDF membranes. After blocked with 5%(ATPsyn-β and β-actin) or 2% fat-free milk(MRP1), the membranes were incubated with antibody against human mitochondrial ATPsyn-β (1∶800 dilutions; Abcam, UK) or MRP1 (1∶400 dilutions; Abcam, UK) or β-actin (1∶5 000 dilutions; Sigma, USA) at 4°C overnight. The bound antibodies were detected using horseradish peroxidase (HRP) - conjugated IgG and visualized with enhanced chemiluminescence (ECL) detection reagents (Thermo scientific, USA).

### Flow cytometry assay of mitochondrial ATPsyn-β protein

Cells were washed twice with PBS and fixed in freshly made 4% paraformaldehyde for 10 min on ice. Fixed cells were permeabilized in PBS containing 0.25% Triton X-100 and 5% fetal calf serum for 5 min, then incubated at 4°C overnight with mouse anti human mitochondrial ATPsyn-β (Abcam, UK) at 1∶100 dilutions. The cells were washed with PBS two times and incubated with FITC-conjugated goat anti-mouse immunoglobulin antibody (Santa Cruz, USA) for 30 min at room temperature in the dark, then detected by flow cytometer.

### Measurement of mitochondrial ATP synthase activity

The mitochondrial fractions from BMMCs of 42 AML patients and cell lines were isolated using a mitochondria isolation Kit (Thermo scientific, USA). The mitochondrial fractions were solubilized in lysis buffer containing 2% CHAPS, sonicated for 10 s, and then centrifuged at 13 000 g, 5 min at 4°C. Supernatant was collected; the protein concentration was measured with a BCA protein assay kit (Thermo Scientific, USA). Then equal amounts of protein were used for the analysis of ATPase/ATP synthase. ATPase/ATP synthase activity was measured in the direction of ATP hydrolysis using an ATPase assay kit (Innova Biosciences). In uncoupled and broken mitochondria, ATP synthase will hydrolyse (rather than synthesize) ATP. The ATP synthase activity in the mitochondria can thus be monitored at saturating ATP levels.

### Cell transfection and RNA interference

For RNA interference studies, 50 nM small interfering RNA (siRNA) duplexes directed targeting ATPsyn-β (sc-40565) or control siRNA (sc-37007) purchased from Santa Cruz Biotechnology were transiently transfected into HL-60 cells using Lipofectamine2000 reagent(Invitrogen, USA) following the manufacturer's recommendations. After 48 h, cells were harvested and western blot was performed to confirm knockdown of ATPsyn-β. The chemo-sensitivity to adriamycin of transfected HL-60 cells was evaluated by MTT assay.

DNA transfection was performed using X-tremeGENE HP reagent (Roche, Germany) according to the manufacturer's protocol. HL-60/ADM cells (5×10^5^/well) were exposed to 3 µg/well of ATPsyn-β expression vector ATPsyn-β pcDNA3.1 (+) and GFP-pcDNA3.1 (+) (Synthesized by Gene-Pharma Company). The effect of transfection was detected 48 h after transfection by western blot. The chemo-sensitivity to adriamycin was evaluated by MTT assay.

### Apoptosis analysis

Cells were treated with 0.5 µM (HL-60 cells) or 10 µM (HL-60/ADM cells) adriamycin for 36–48 hours. The apoptotic morphology was evaluated by Hoechst 33258 staining (KeyGen Biotech, China) and Wright-Giemsa staining. Cells apoptotic ratio was assessed with an Annexin V-APC apoptosis detection kit (KeyGen Biotech, China) after different concentrations of adriamycin treatment.

### Statistical analysis

Results were analyzed using the SPSS 17.0 software (IBM, Chicago, IL) with the two-sided P value less than 0.05 as statistically significant. Difference between values of two groups was performed by student's t-test. The correlations between ATPsyn-β expression and IC_50_ of BMMCs to adriamycin, ATPsyn-β expression and ATP synthase activity were assessed using Pearson's correlation analysis. ATPsyn-β mRNA expression was divided into high or low group by the cutoff point showing 1.66(the mean value of ATPsyn-β mRNA expression of total 110 AML patients). Survival curves were derived from Kaplan-Meier estimates and compared by log-rank test. Complete remission (CR) duration was calculated from the time of CR until relapse. Overall survival (OS) was measured from the time of diagnosis until the date of death of any cause or last follow-up.

## Results

### Mitochondrial ATPsyn-β expression profiling and ATP synthase activity in HL-60/ADM cells

**Figure 1:**
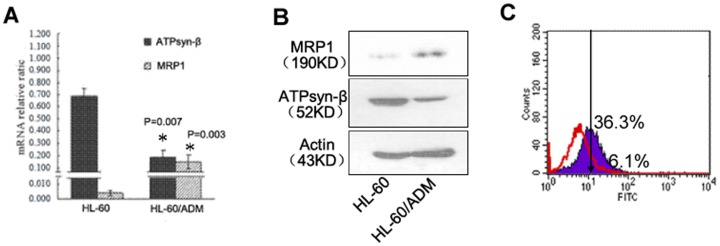
Analysis of mitochondrial ATPsyn-β in HL-60 and HL-60/ADM cell lines. A. Loss of mitochondrial ATPsyn-β mRNA and gain of MRP1 mRNA in HL-60/ADM cells. Columns, means of three experiments; bars, SD. B. Western blot detection of mitochondrial ATPsyn-β and MRP1 protein. β-actin is used as loading control. Data represent one of three repeats. C. Flow cytometric histogram of mitochondrial ATPsyn-β expression from HL-60/ADM cells (red line, 6.1%) and HL-60 cells (purple color, 36.3%).

To evaluate the expression profiling of mitochondrial ATPsyn-β in HL-60 and HL-60/ADM cells, real-time quantitative PCR, western blot analysis and flow cytometry were performed. Consistent with diminished mRNA expression, mitochondrial ATPsyn-β protein exhibited a significant decrease in HL-60/ADM cells compared to HL-60 cells (Figure 1). Compared with HL-60 cells, mitochondrial ATPsyn-β protein expression was 2.2-fold lower in HL-60/ADM cells, while MRP1 protein was 3.4-fold higher in HL-60/ADM cells, suggesting that mitochondrial ATPsyn-β and MRP1 were closely related and involved in the development of MDR. It is interesting to note that HL-60/ADM cells displayed an evident lower mitochondrial ATPase activity(38.2±2.0 U) than HL-60 cells (132.1±14.7 U), which indicated that down-regulated expression of mitochondrial ATPsyn-β and decreased ATPase activity in HL-60/ADM cells were both associated with drug resistance of leukemia cells.

### Silencing mitochondrial ATPsyn-β gene leads to increased chemo- or apoptotic-resistance in HL-60 cells and over-expression of mitochondrial ATPsyn-β increases chemo-sensitivity and promotes apoptosis in HL-60/ADM cells

**Figure 2:**
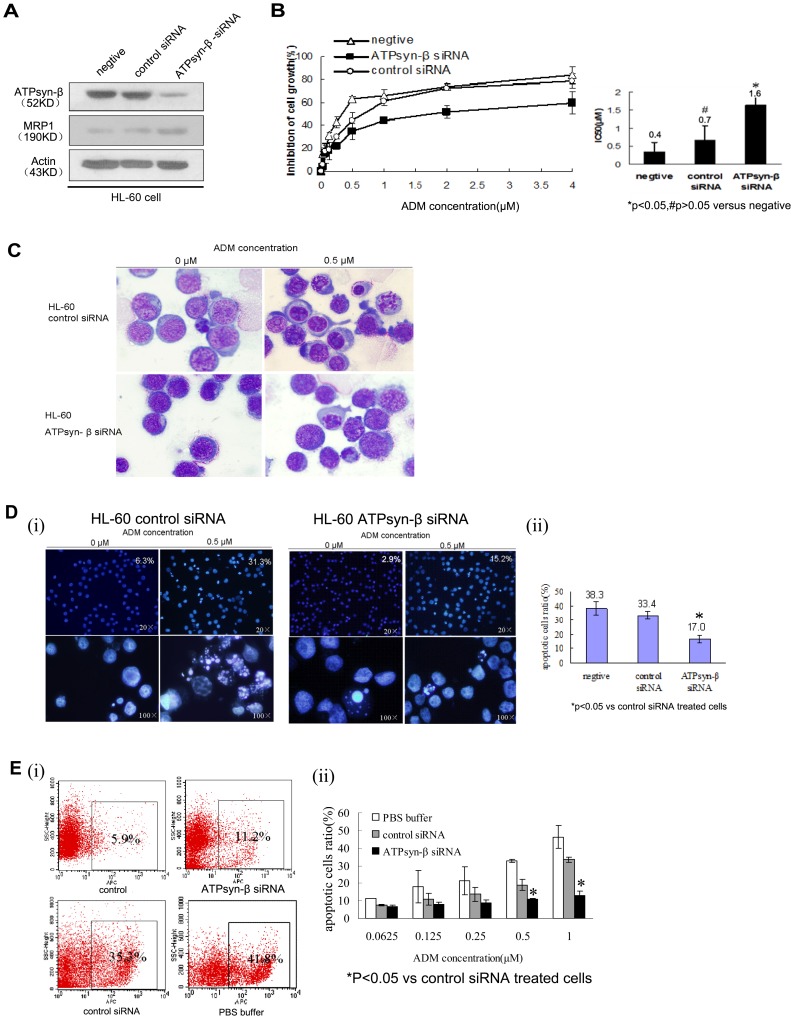
Effect of ATPsyn-β siRNA on adriamycin sensitivity in HL-60 cells. A. 48-β and MRP1 were determined by western blot. Results are representative of three repeats. B. Inhibition percentages of cell proliferation in various concentrations of adriamycin-treated HL-60 cells with or without siRNA transfection (0.0625, 0.125, 0.25, 0.5, 1, 2, 4 µM, for 48 h) determined by MTT assay. The values or Columns represent mean±SD of triplicate experiments. C. Morphologic change in HL-60 cells transfected with ATPsyn-β siRNA. 48 h after transfection, HL-60 cells were treated with 0.5 µM adriamycin, and cultured for 48 hours. Then cells were stained with Wright-Giemsa and photographed (×1000). D. Morphological features for apoptosis in control siRNA or ATPsyn-β siRNA-treated HL-60 cells were revealed by Hoechst 33258 staining (×200, up panel; ×1000, below panel). After transfection, cells were treated with 0.5 µM adriamycin for 48 hours. Condensed chromatin and apoptotic body could be found in control siRNA-treated HL-60 cells by fluorescence microscope (Olympus). For each Hoechst experiment at least 200 cells in 5 random scope fields were counted for apoptotic rate. The mean percentage of apoptotic cells treated with ATPsyn-β-siRNA was 17.0%, which was significantly lower than cells transfected with nonsilencing control siRNA (33.4%). Experiments in this figure were repeated twice and similar results were obtained. E. Percentages of apoptotic cells in adriamycin-treated HL-60 cells after siRNA transfection based on annexin V-APC expression assays. HL-60 cells with indicated treatment (PBS buffer, control siRNA, ATPsyn-β siRNA) were incubated with different dose of adriamycin(0.0625, 0.125,0.25,0.5,1 µM) for 36 h. (i) Representative flow cytometry results(adriamycin = 1 µM). The percentage of apoptotic HL-60 cells transfected with mitochondrial ATPsyn-β siRNA was 11.5%, which was significantly lower than the percentage of apoptotic cells transfected with control siRNA (35.3%) and PBS buffer only (41.9%). (ii) Data are presented as the mean ± SD for three independent experiments. *P<0.05 vs control siRNA treated cells.

To further explore a causal relationship of mitochondrial ATPsyn-β and adriamycin resistance, HL-60 cells were transfected with siRNA specific to ATPsyn-β gene, and cell viability was analyzed after different concentrations of adriamycin treatment by MTT assay. Following knockdown of ATPsyn-β gene, a decreased adriamycin sensitivity in HL-60 cells was observed, the IC_50_ was 1.7 µM, compared to 0.6 µM in control siRNA transfected HL-60 cells and representing a 2.6-fold decrease towards drug sensitivity (Figure 2B). Then we evaluated the effect of apoptotic sensitivity to adriamycin by Hoechst 33258 staining, Wright-Giemsa staining and Annexin V-APC assay. Wright-Giemsa staining provided clearly the evidence of apoptotic morphology in HL-60 cells (Figure 2C); The Hoechst 33258 staining showed that the mean percentage of apoptotic cells in ATPsyn-β-siRNA group was 17.0%, which was lower than the percentage of apoptotic cells in control group (33.4%)(Figure 2D). The mean ratio of apoptotic cells in ATPsyn-β siRNA group by Annexin V assay was also lower than those transfected with control siRNA (Figure 2E). These findings suggested that mitochondrial ATPsyn-β played an important role in regulating adriamycin induced apoptosis of HL-60 cells and was involved in the adriamycin resistance of leukemia cells.

**Figure 3:**
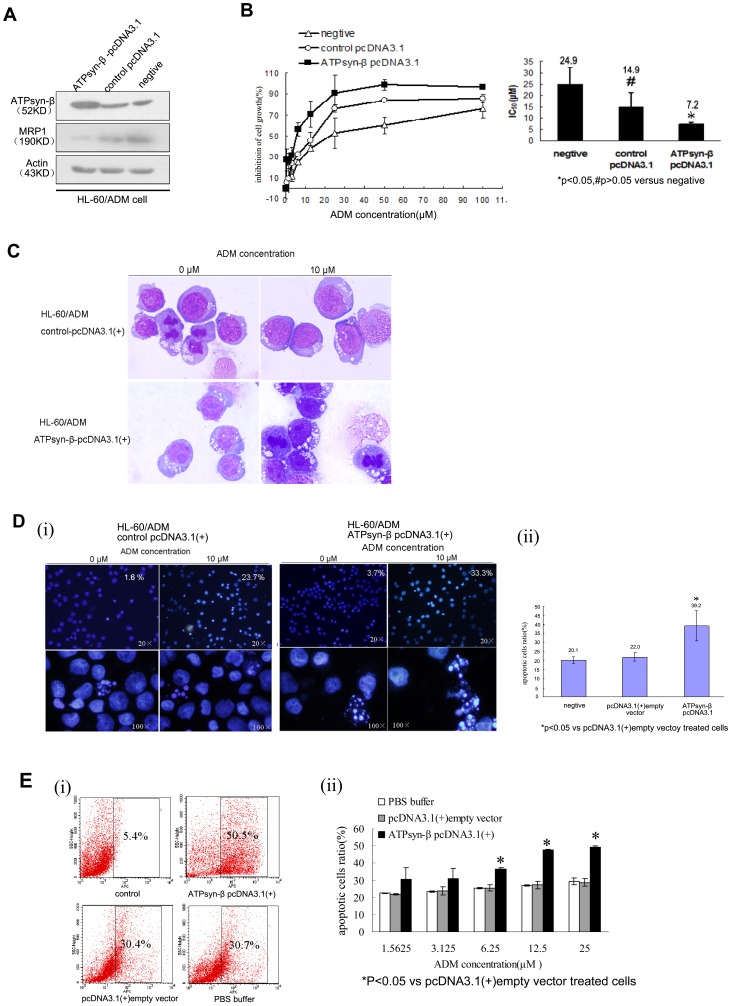
Effect of ATPsyn-β recombinant plasmid on adriamycin sensitivity in HL-60/ADM cells. A. 48-β and MRP1 were determined by western blot. Results are representative of three repeats. β-actin is used as loading control. B. Inhibition percentages of cell proliferation in various concentrations of adriamycin-treated HL-60/ADM cells after ATPsyn-β-pcDNA3.1(+) transfection (1.5625, 3.125, 6.25, 12.5, 25, 50, 100 µM, for 48 h) determined by MTT assay. The values or Columns represent mean±SD of triplicate experiments. C. Morphologic change in HL-60/ADM cells transfected with ATPsyn-β-pcDNA3.1 (+). 48 h after transfection, HL-60/ADM cells were treated with 10 µM adriamycin, and cultured for 48 hours. Then cells were stained with Wright-Giemsa and photographed (×1000). D. Morphological features for apoptosis in control pcDNA3.1 (+) or ATPsyn-β pcDNA3.1 (+)-treated HL-60/ADM cells were revealed by Hoechst 33258 staining (×200, up panel; ×1000, below panel). After transfection, cells were treated with 10 µM adriamycin for 48 hours. For each Hoechst experiment at least 200 cells in 5 random scope fields were counted for apoptotic rate. The mean percentage of apoptotic cells treated with ATPsyn-β-pcDNA3.1 (+) was 33.3%, which was higher than the percentage of apoptotic cells transfected with empty control pcDNA3.1(+) (23.7%). Experiments in this figure were repeated twice and similar results were obtained. E. Percentages of apoptotic cells in adriamycin-treated HL-60/ADM cells after plasmid transfection based on annexin V-APC expression assays. HL-60/ADM cells with indicated treatment (PBS buffer, pcDNA3.1(+) empty vector, ATPsyn-β pcDNA3.1(+)) were incubated with different dose of adriamycin(1.5625, 3.125, 6.25, 12.5, 25 µM) for 36 h. (i) Representative flow cytometry results(adriamycin = 25 µM). (ii) Data are presented as mean ± SD for three independent experiments. *P<0.05 vs pcDNA3.1 (+) empty vector treated cells.

Besides, a mitochondrial ATPsyn-β over-expression cell line, HL-60/ADM-R, was established by transfection with ATPsyn-β-pcDNA3.1 (+) plasmid in HL-60/ADM cells. Compared to HL-60/ADM cells, obvious elevation of mitochondrial ATPsyn-β expression was detected in HL-60/ADM-R cells by western blot (Figure 3A). Gene transfection also led to decreased adriamycin resistance in HL-60/ADM-R cells and the IC_50_ was 7.2 µM, compared to that of 14.9 µM in cells with empty plasmid transfection, representing a 2.1-fold increase towards drug sensitivity (Figure 3B). To examine the effect of mitochondrial ATPsyn-β on chemotherapeutic agent induced apoptosis, HL-60/ADM-R cells and corresponding control cells were incubated with 10 µM adriamycin for 36 or 48 hours, followed by cell morphology, Hoechst 33258 staining and Annexin V assays. HL-60/ADM-R cells exhibited increased number of apoptotic cells and typical apoptotic morphologic changes, in which the mean ratio of apoptotic cells was 39.2%, which was significantly higher than that of control cells (22.0%) (Figure 3D). Consistent with morphology data, the percentage of apoptotic cells in HL-60/ADM-R cells detected by Annexin V assay was also obviously increased (Figure 3E). These results strongly suggested that up-regulation of mitochondrial ATPsyn-β could reverse adriamycin resistance and regulate the apoptosis pathway in HL-60/ADM cells.

We also examined the expression of MRP1 before and after transfection. The results documented that up-regulation of ATPsyn-β decreased MRP1 expression, and in contrast, inhibition of ATPsyn-β expression with siRNA was able to increase MRP1 level, indicating there was an intrinsic relationship between mitochondrial ATPsyn-β and MRP1.

### Therapeutic response of AML patients is consistent with adriamycin resistance phenotype of primary leukemia cells in vitro

We evaluated adriamycin chemosensitivity of BMMCs from 80 AML patients (relapsed/refractory n = 42, remission n = 38). The mean IC_50_ to adriamycin was 13.7 µM in relapsed/refractory samples, which was 7.8-fold higher than that in remission patients (1.6 µM).These data confirmed a resistance to adriamycin in primary leukemia cells from relapsed/refractory AML patients in vitro.

### Expression of mitochondrial ATPsyn-β in primary leukemia cells from AML patients

**Figure 4:**
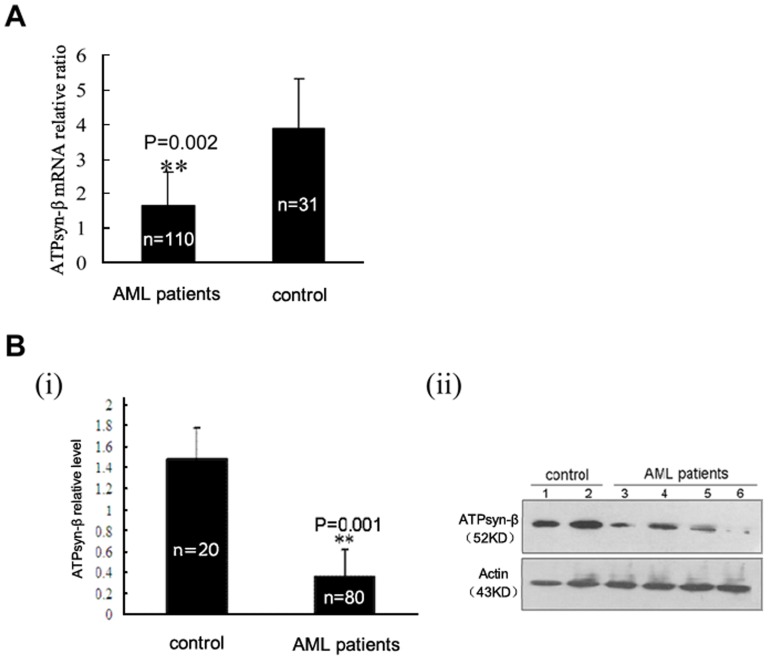
Mitochondrial ATPsyn-β expression in primary leukemia cells. A. Downregulation of ATPsyn-β mRNA in primary AML samples. Columns, means of primary samples; bars,SD. B. ATPsyn-β protein expression determined by western blotting. β-actin is used as loading control.(i) Columns, means of gray-scale value of primary AML samples or control samples; Error bars, SD. (ii) Representative results. Lane 1–2, control samples; lane 3–6, AML samples.

We analyzed the expression levels of mitochondrial ATPsyn-β mRNA and protein in primary bone marrow cells from AML patients and healthy individuals. Our results showed that the level of mitochondrial ATPsyn-β mRNA in AML patients (n = 110) was obviously reduced (p<0.01) (Figure 4A). In accordance with the change of mRNA, western blot analysis documented that mitochondrial ATPsyn-β protein in AML samples (n = 80) was also significantly decreased (p<0.01) (Figure 4B) when compared with those in control individuals (n = 20), suggesting down-regulated expression of mitochondrial ATPsyn-β in AML.

**Figure 5:**
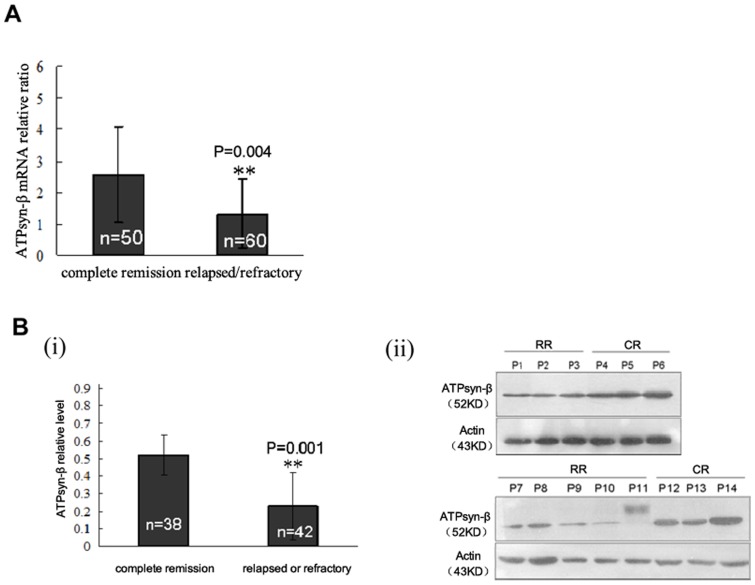
Down-regulation of mitochondrial ATPsyn-β in bone marrow mononuclear cells from relapsed/refractory AML patients. A. Downregulation of ATPsyn-β mRNA in relapsed/refractory AML samples. Columns, means of primary samples; bars, SD. B. ATPsyn-β protein expression determined by western blotting. β-actin is used as loading control.(i) Columns, means of gray-scale value of remission AML samples or relapsed/refractory samples; Error bars, SD. (ii) Representative results. CR: complete remission; RR: relapsed/refractory.

**Figure 6:**
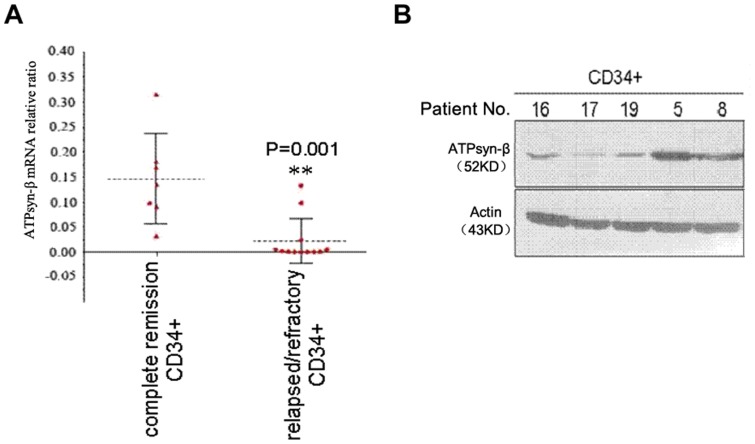
Down-regulation of mitochondrial ATPsyn-β in CD34+ cells from relapsed/refractory AML patients. A. Loss of ATPsyn-β mRNA in relapsed/refractory AML samples. The dotted line indicates means of primary samples; bars, SD;n = 20. B. Mitochondrial ATPsyn-β protein expression (n = 5). No. 16, 17, 19, relapsed/refractory samples; No. 5, 8, presentation samples from remission samples.

We further stratified AML patients according to their therapeutic response and explored the relationship between mitochondrial ATPsyn-β expression and clinical stages both in BMMCs (mRNA assay: n = 110; western blot assay: n = 80) (Figure 5) and in CD34^+^ cells (n = 20) (Figure 6). We found that the transcript level of mitochondrial ATPsyn-β was evidently lower in relapsed/refractory AML samples(n = 60) than that in remission samples (n = 50), which was consistent with protein level results, suggesting that suppressed expression of mitochondrial ATPsyn-β was associated with the development of drug resistance in AML patients.

### Decreased expression of mitochondrial ATPsyn-β is associated with adriamycin resistance of leukemia cells

**Figure 7:**
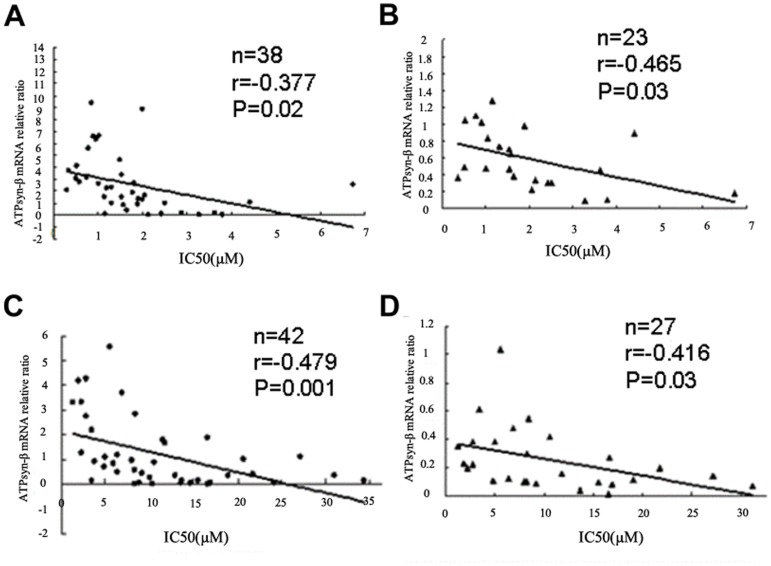
Correlation of mitochondrial ATPsyn-β expression and IC_50_ of AML primary samples to adriamycin in vitro. ATPsyn-β mRNA (A) and protein expression (B) showed an inverse correlation with IC_50_ of primary cells to adriamycin from remission patients. ATPsyn-β mRNA (C) and protein expression (D) showed an inverse correlation with adriamycin IC_50_ of primary cells from relapsed/refractory AML patients.

Subsequent correlation analysis revealed that the mRNA and protein levels of mitochondrial ATPsyn-β were both inversely correlated with the IC_50_ values of adriamycin in relapsed/refractory AML samples and presenting samples who went into remission duration (p<0.05) (Figure 7). These results further documented the fact that downregulation of mitochondrial ATPsyn-β was associated with drug resistance in AML patients.

### Low activity of mitochondrial ATPase exists in primary leukemia cells from relapsed/refractory AML patients

We measured mitochondrial ATPase activity in primary leukemia cells from 42 AML patients (relapsed/refractory n = 26, complete remission n = 16) and compared the difference between the two groups. The results showed that the mean mitochondrial ATPase activity in relapsed/refractory AML patients was significantly reduced (20.3±11.9 U) when compared with that of complete remission patients (31.2±14.6 U) (p<0.05). Further analysis indicated that mitochondrial ATPase activity of AML patients positively correlated with the mRNA levels of ATPsyn-β(r = 0.29, P = 0.03).

### Correlation between mitochondrial ATPsyn-β mRNA level and chemotherapeutic efficacy in AML patients

**Figure 8:**
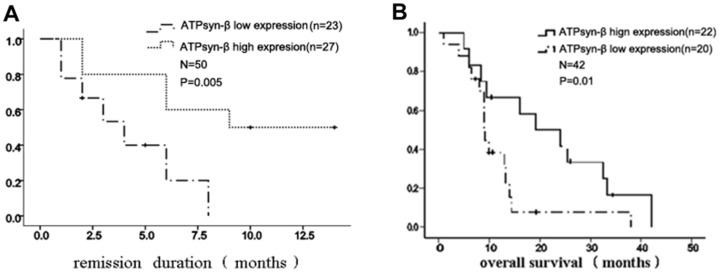
Correlation between mitochondrial ATPsyn-β gene and response to chemotherapy. A. Kaplan–Meier survival analysis showed that AML patients in remission (n = 50) with low expression of ATPsyn-β mRNA at diagnosis had shorter CR1 duration. B. Kaplan–Meier survival analysis showed that relapsed/refractory AML patients (n = 42) with low expression of ATPsyn-β mRNA had shorter overall survival.

Kaplan-Meier survival analysis was performed in 80 AML patients (complete remission: n = 38, relapsed/refractory: n = 42). Based on clinical and follow up data after the completion of induction chemotherapy, the results showed that remission duration patients (n = 38) with low expression of ATPsyn-β mRNA at diagnosis had shorter CR1 remission duration (6.8±2.5 months vs. 10.4±1.4months). Relapsed/refractory AML patients with low ATPsyn-β mRNA expression had a shorter overall survival (10.7±7.9 months vs. 21.3±1.3 months) (Figure 8).

## Discussion

In recent years, energy metabolism of tumors has been recognized as an additional phenotypic trait of cancer cell with the Warburg effect as the flagship leading the conceptual framework. Mitochondrion plays an essential role in cellular energetic metabolism [31] and the execution of cell death [32]. Both the activity of oxidative phosphorylation [33], [34] and the subunits of ATP synthase [35] are required for the execution of apoptosis and energetic metabolism that the two mitochondrial activities are molecularly and functionally integrated. The mitochondrial ATP synthase is a master regulator of energetic metabolism. It has been consistently documented that the expression of H^+^-ATP synthase β subunit was down-regulated in most prevalent human carcinomas and might be a “bioenergetic signature” [13], [15], [17], [19]. The bioenergetic signature not only represents the prognostic significance in breast [17], [19], colon [15], [22] and lung cancer patients [13], [18], but also reflects the mechanism of MDR to chemotherapy in some solid tumors and CML [25]. More recently, a fundamental relationship between mitochondrial bioenergetics and tumor response to cytotoxic chemotherapy has been documented [36], which is consistent with the fact that a specific repression of ATPsyn-β expression in cancerous cell contributes to the growth of tumor and cancer progression.

We have previously demonstrated that down-regulation of mitochondrial ATP synthase (ATPsyn-β) is associated with adriamycin resistance in CML patients [25]. In this study, we investigated the expression of mitochondrial ATPsyn-β in primary leukemia cells from 110 AML patients and its potential association with adriamycin resistance phenotype. Consistent with chemo-resistance phenotype in vitro, our data indicated that the expression of mitochondrial ATPsyn-β, both at the protein level and at the mRNA level in CD34^+^ or BMMCs from relapsed/refractory patients was significantly lower than from those remission samples. Although the case number of each group was inconsistent, these results strongly suggested a lower expression of mitochondrial ATPsyn-β in AML patients. The changes of bioenergetic signature-mitochondrial ATPsyn-β may be involved in the MDR at leukemia stem cell level. More importantly, we revealed that there was indeed an intrinsic relationship between adriamycin resistance and mitochondrial ATPsyn-β down-regulation.

It has been demonstrated that overexpression of ATPase inhibitor factor 1(IF1) in human carcinomas is able to inhibit the activity of ATP synthase [36], [37], the main mechanism is exerted at the level of translation [38]. In order to verify the above observation, we carried out the activity assay on ATPase activity in primary leukemia cells. The ATPase activity in relapsed/refractory AML patients was significantly lower than that in AML patients with CR and the changes were parallel with mitochondria ATPsyn-β mRNA level. Likewise, dysregulation of ATPsyn-β, accompanied by defect in mitochondrial ATPase activity was also linked to chemotherapy resistance. These results further strengthen our previous conclusion.

Mitochondrial ATPsyn-β expression as a bioenergetic signature has been shown to be a therapeutic response marker in different cancer cell lines, both for mono and combined chemotherapy [15], [21]–[24], [39], [40]. Two independent studies in large cohorts of colon cancer patients indicated that low tumor expression of ATPsyn-β was associated with poor overall and disease-free survival of the patients [13], [22]. Further studies showed that the bioenergetic signature of isogenic colon cancer cells predicted the cell death response to treatment with 3-bromopyruvate, iodoacetate or 5-fluorouracil (5-FU). To reveal whether there was a relationship between mitochondrial ATPsyn-β expression and clinical outcome of AML, we performed a survival analysis. Our results indicated that the mRNA levels of ATPsyn-β positively correlated with the response to induction chemotherapy. It was found that low ATPsyn-β mRNA expression correlated with a shorter first remission duration in newly diagnosed patients and revealed both a worse prognosis and a shorter overall survival (OS) in relapsed/refractory patients, which suggested that ATPsyn-β may be a valuable parameter for prognosis assessment in AML patients.

Our results showed that up-regulation of mitochondrial ATPsyn-β in leukemia cells led to significantly increased sensitivity to adriamycin-induced cell growth inhibition and decreased resistance to adriamycin-triggered apoptosis. Besides, siRNA-mediated silencing of ATPsyn-β in HL-60 cells decreased its sensitivity to adriamycin and exhibited an obvious apoptotic-resistance. This observation strongly supports the fact that mitochondrial ATPsyn-β may be a single molecular event to regulate the response to chemotherapy in human acute myeloid leukemia cells; repression of ATPsyn-β ensue leukemia cells with an adriamycin-resistant phenotype. It has been documented that treatment of HCT116 colon cancer cells with specific inhibitor of the ATP synthase oligomycin, resulted in cells with down-regulated expression of ATPsyn-β and inhibit or delayed the execution of death. Conversely, treatment of HCT116 cells with 2-DG, an inhibitor of glycolysis, resulted in cells displaying up-regulated expression of ATPsyn-β, which has been shown to be particularly effective in impairing cancer growth [14]. Besides, Schulz TJ et al reported that by overexpressing rate-limiting mitochondrial proteins, tumor growth could be efficiently reduced in nude mice [41]. A probable explanation for down-regulated mitochondrial ATP synthase and apoptotic resistance is that suppression of mitochondrial ATPsyn-β damages the overall oxidative phosphorylation capability of the cell, thus, an abrogated mitochondrial activity contributes to a diminished potential for ROS signaling which engage in mitochondrial mediated cell death [14], [21], [33], [34].

It has been shown that some molecular components that participate in oxidative phosphorylation, such as Cyt-c (cytochrome c), AIF(apoptosis inducing factor) and mitochondrial synthase are required for the efficient execution of cell apoptosis [35]. In apoptosis, a sufficient supply of ATP is necessary to trigger the activation of caspases family and apoptosome formation [42]. Ni Chonghaile et al [43] found that pretreatment mitochondrial priming with peptides derived from pro-apoptotic BH3 domains of proteins may decrease the apoptotic threshold of mitochondria in tumor cells and enhance the efficacy of cytotoxic agents, suggesting mitochondria modification, even ATP supplement, may be a new tactics for overcoming chemotherapy resistance of tumor cells. Another explanation for mitochondrial ATPsyn-β mediated multidrug resistance is MRP1. MRP1/ABCC1 contributes to several physiological functions and pathophysiological incidents, such as inflammatory responses and oxidative stress [44]. MRP1 was firstly discovered in a multidrug-resistant small-cell lung cancer cell line [45]. Afterward the increased expression of MRP1 has been reported in a variety of hematological diseases. There is evidence that MRP1 may contribute to the lower intracellular daunorubicin concentration [46] and is correlated with poor treatment outcome [47]. However, the exact mechanism and clinical application potential needs to be further elucidated in future studies.

## Conclusion

Our study demonstrates that mitochondrial ATPsyn-β expression is dysregulated and ATP synthase activity is reduced in AML, which clearly contribute to multidrug resistance of leukemia cells. We also find that reduced ATPsyn-β expression is an adverse prognostic factor correlated with chemotherapy resistance. Importantly, our study provides a potential approach to reverse multidrug resistance in AML treatment by mitochondria modification. Targeting energetic metabolism might present an alternative leukemia treatment strategy in the near future.
